# Wie wir dort gelandet sind, wo wir jetzt wegmüssen: Geschichte und Zukunft des deutschen Wissenschaftssystems

**DOI:** 10.1007/s00048-024-00398-x

**Published:** 2024-08-05

**Authors:** Amrei Bahr, Kristin Eichhorn, Sebastian Kubon

**Affiliations:** 1 Institut für Philosophie, Universität Stuttgart, Seidenstraße 36, 70174 Stuttgart, Deutschland; 2 Institut für Literaturwissenschaft, Abteilung Neuere Deutsche Literatur I, Universität Stuttgart, Keplerstr. 17, 70174 Stuttgart, Deutschland; 3 Historisches Seminar, Mittelalterliche Geschichte, Ludwig-Maximilians-Universität München, Geschwister-Scholl-Platz 1, 80539 München, Deutschland

Zur Wissenschaftsgeschichte gehört immer auch ein Blick auf die Geschichte der Rahmenbedingungen, unter denen wissenschaftliche Arbeit stattfindet. Seit 2021 ist das Thema prekäre Arbeit in der Wissenschaft durch den Hashtag #IchBinHanna öffentlich etabliert: Begonnen als Twitter-Initiative in Reaktion auf ein missglücktes Video des Bundesministeriums für Bildung und Forschung (BMBF) aus dem Jahr 2018, das anhand einer animierten Figur namens Hanna das Wissenschaftszeitvertragsgesetz (WissZeitVG) erklärte und dabei die umfassenden Sonderbefristungsregelungen in der Wissenschaft mit abfälligen Formulierungen als vorgebliche Generationengerechtigkeit rechtfertigte („damit nicht eine Generation alle Stellen verstopft“),^1^ ist #IchBinHanna inzwischen aus der politischen und öffentlichen Debatte nicht mehr wegzudenken. Bereits aus der Vorgängeraktion #95vsWissZeitVG – einer Sammlung von 95 Thesen gegen das WissZeitVG, entstanden am Reformationstag 2020 – ist ein Sammelband hervorgegangen (Bahr et al. 2021a); das Buch zu #IchBinHanna folgte 2022 (Bahr et al. 2022).^2^ Seit der Bundestagswahl 2021 arbeitet das BMBF an einer Reform des Sonderbefristungsrechts in der Wissenschaft und hat bereits einen Referentenentwurf hervorgebracht (Sozialdemokratische Partei Deutschlands et al. 2021). Der Reformprozess ist indes keinesfalls abgeschlossen, da es über zentrale Fragen noch Streitpunkte gibt.

Natürlich ging es sowohl bei #95vsWissZeitVG als auch bei #IchBinHanna von Anfang an um mehr als nur das WissZeitVG. Vielmehr ist es uns gelungen, eine grundsätzliche Debatte darüber anzustoßen, unter welchen Prämissen Wissenschaft in Deutschland stattfindet und sinnvollerweise stattfinden sollte. Denn die ganze Befristungsdebatte offenbart letztlich ideologische Vorannahmen, die schon allein wegen ihrer mangelnden wissenschaftlichen Fundiertheit dringend ad acta gelegt werden müssen. Dazu gehört die auch im Hanna-Video zentrale Leitthese, Innovation bedürfe der ständigen (Personal‑)Fluktuation, die aber nicht belegt ist (Kubon 2021: 12–32). Überdies blendet dieses Dogma die zahlreichen negativen Effekte von zu viel Personalwechsel aus, die inzwischen auch finanziell für Wissenschaft und Gesellschaft ein folgenschweres Minusgeschäft geworden sind: Transaktionskosten und der Verlust von (*tacit*) knowledge. Eine Studie des Bayerischen Staatsinstituts für Hochschulforschung und Hochschulplanung im MINT- und wirtschaftswissenschaftlichen Bereich hat ergeben, dass insbesondere die ständige Fluktuation gegenwärtig als das wesentliche Innovationshemmnis gesehen wird (Ostmeier & Welpe 2021). Längerfristige Grundlagenforschung sei unter diesen Bedingungen kaum noch zu leisten.

Einzelne Wissenschaftler:innen haben keine Zeit für Forschung, weil sie vom ersten Tag ihres oftmals knapp befristeten Vertrags an damit beschäftigt sind, sich für die nächste Stelle zu bewerben. Ständig müssen Anträge geschrieben und Mittel akquiriert werden. Dafür muss Forschung in „Häppchen“ aufgeteilt und so präsentiert werden, dass sie die Begutachtung besteht. Dies bedingt ein Mainstreaming und eine thematische Verengung von Forschungsansätzen; es wird nicht mehr das erforscht, was nötig ist, sondern das, was Aussicht auf Förderung hat. Und nicht zuletzt: Eine wissenschaftliche Laufbahn ist so unsicher und mit so vielen strukturellen Hürden verbunden, dass sie viele Personengruppen von vornherein aus der vermeintlichen „Bestenauslese“ ausschließt. Man muss sich Wissenschaft leisten können, genügend finanzielle Ressourcen mitbringen, frei von familiären Verpflichtungen und körperlichen Einschränkungen sein. Auch herkunftsbezogene strukturelle Benachteiligung ist durch die Art und Weise, wie das Wissenschaftssystem aktuell funktioniert, eher die Regel denn die Ausnahme. Keine Bestenauslese ist die Folge, sondern eine „Verbliebenenauslese“.

## Der Blick zurück: Wo die heutigen Diskurse herkommen

Man kann den Weg, der zur heutigen – wirklich desolaten – Lage geführt hat, in Ansätzen nachverfolgen, wenn man die historischen Unterlagen sichtet, soweit ihr Zugang archivrechtlich nicht beschränkt ist (Domke 2019). Die Diskussion über Teilzeit und Befristung, wie wir sie in unserem Buch von 2022 rekonstruiert haben, erweist sich jedoch bereits als sehr aufschlussreich. So sprach sich der 1957 gegründete Wissenschaftsrat in den 1960er Jahren für eine Zunahme der Stellen für wissenschaftliches Personal aus, um auf die gestiegenen Studierendenzahlen und die Überarbeitung der beschäftigten Wissenschaftler:innen – vor allem in der Qualifikationsphase – zu reagieren. Aus dieser Problemanalyse entstand in der zweiten Hälfte der 1970er Jahre ein neues Narrativ, das bis heute unser Wissenschaftssystem prägt: Letztlich gehe es in der Wissenschaft darum, so vielen Personen wie möglich die Gelegenheit zur Qualifikation zu geben.^3^ Deshalb gelte es, im konkreten Einzelfall die „Verweildauer“ der Beschäftigten in der Wissenschaft zu begrenzen. Dementsprechend schlug der Wissenschaftsrat 1980 den verstärkten Einsatz von Teilzeitstellen vor – erstens, um mehr Personen eine „Chance“ gewähren zu können, zweitens, weil eine Auslagerung der Qualifikation aus der Arbeits- in die Freizeit eine Entlastung der Beschäftigten zur Folge habe: „Da bei voller Inanspruchnahme durch Dienstleistungen nur wenig Zeit für die eigene Weiterqualifizierung zur Verfügung steht, gewinnt der Beschäftigte Zeit für seine Weiterqualifizierung, wenn er in einem Teilzeitbeschäftigungsverhältnis steht“ (Wissenschaftsrat 1980: 18). Dies freilich dürfe nur in den Fachbereichen getan werden, wo kein Personalmangel herrsche; in „Fächergruppen, in denen qualifizierte Mitarbeiter anders nicht gewonnen werden können“, müssten hingegen „die Möglichkeiten zur Vollzeitbeschäftigung“ (ebd.) ausgeschöpft werden.

Damit kam aus der Wissenschaft selbst eine verquere Logik in den Diskurs, die von einer auf eine Marktliberalisierung auch der Hochschulen setzenden Politik dankbar aufgegriffen wurde: 1985 gab es das erste Gesetz über befristete Arbeitsverträge mit wissenschaftlichem Personal an Hochschulen und Forschungseinrichtungen (HFVG), das eine standortspezifische Befristungshöchstdauer von fünf Jahren festlegte, unter der Voraussetzung eines regelmäßigen Arbeitgeber:innenwechsels – was in der Praxis recht umfassende Befristungsmöglichkeiten schuf (vgl. Bahr et al. 2022: 40 f.). Damit war in weiten Teilen die Grundlage für die meisten Probleme gelegt, unter denen das heutige deutsche Wissenschaftssystem leidet und die im Zentrum der #IchBinHanna-Debatte stehen. Wissenschaft sei eine Beschäftigung, die der eigenen „Qualifikation“ und Selbstverwirklichung diene. Die Leistung, die Wissenschaftler:innen auch jenseits der Professur für ihre Institutionen und die Gesamtgesellschaft erbringen (und zwar nicht zuletzt in Form ihrer Arbeit an Promotions‑, Habilitations- und Postdoc-Projekten, die entgegen der vermeintlichen Eigennützigkeit sehr wohl einen genuinen Mehrwert für die Wissenschaft bedeuten), gerät damit aus dem Blick.

In der Folge hat sich die Art und Weise, wie wir Wissenschaft betreiben, so stark verändert, dass sie letztlich eine Entfernung von wissenschaftseigenen Standards und Kriterien mit sich bringt. Ob ein bestimmtes Forschungsvorhaben durchgeführt wird oder nicht, hängt inzwischen weniger davon ab, ob es denen, die es beabsichtigen, inhaltlich notwendig erscheint. Stattdessen stehen Wissenschaftler:innen unter einem enormen Anpassungsdruck. So sind sie gehalten, Drittmittel einzuwerben und ihre Forschung so zu profilieren, dass sie sie in ihrer individuellen Karriereentwicklung voranbringt – also ihre Chancen auf eine neue Anstellung erhöht. Entsprechend setzt man stärker auf Themen und Methoden, die in dieser Hinsicht risikoärmer, von der Fachcommunity bereits akzeptiert sind oder für die schlicht aufgrund aktueller gesellschaftlicher Trends leichter Fördergelder zu bekommen sind.

Diese Praxis stellt eine klare Gefahr für die Wissenschaftsfreiheit dar.^4^ Sie führt zu einer Verschwendung von Ressourcen, die sich Deutschland eigentlich überhaupt nicht leisten kann: Forschung wird in „Häppchen“ aufgespalten, um möglichst viele kleine Drei-Jahres-Projekte zu ergeben und viele Publikationen zu generieren (Bahr et al. 2021b). Das heißt auch, dass Projekte, die eigentlich aus mehreren „Häppchen“ bestehen müssten, um sinnvoll abgeschlossen werden zu können, vor einem Problem stehen, wenn der Versuch, für weitere „Häppchen“ eine Finanzierung einzuwerben, scheitert. Die Anreize für „neue“ Vorhaben (das einschlägige Buzzword lautet „Innovation“) sind groß; für eine nachhaltige Implementierung und Nutzbarmachung von Forschungsergebnissen in der Breite fehlt jedoch in der Regel das Geld, sodass aufgebaute Datenbanken, programmierte Apps und angeschaffte Geräte meist nach einiger Zeit nicht mehr im Gebrauch sind. Die Steuergelder sind indes geflossen und in dieser Weise verschwendet worden, ohne dass die Gesellschaft den intendierten Nutzen davon erhält. Eine solche „Strohfeuer-Forschung“ zeigt in aller Regel nur selten nachhaltige Effekte.

Gleiches gilt für die im Hanna-Video als so notwendig bezeichnete Personalfluktuation. In der Praxis bedeutet sie, dass die Wissenschaft ständig hochqualifizierte Expert:innen ausbildet, um sie, wenn sie am Peak ihrer Expertise angelangt sind und ihre Fähigkeiten gewinnbringend einsetzen könnten, zum Berufswechsel in Branchen zu zwingen, in denen diese Fähigkeiten nur punktuell gebraucht werden. Stattdessen kommt neues Personal an die Hochschulen, das sich permanent einarbeiten muss. Spätestens in Zeiten des Fachkräftemangels jedoch, in denen es zunehmend schwieriger wird, auch wissenschaftliche Stellen zu besetzen,^5^ gefährden die derzeitigen Rahmenbedingungen das Projekt Wissenschaft als Ganzes. Hochschulen und Forschungseinrichtungen müssen etwas tun, um ihr qualifiziertes Personal zu halten, statt so zu tun, als generiere man Chancen, wenn man Arbeitnehmerschutzregelungen umgehe.^6^

## Der Blick in die Zukunft: Was es braucht, um ein wissenschaftsadäquateres System zu schaffen

Die Komplexität der oben beschriebenen Situation macht es notwendig, an diversen Stellen neu anzusetzen. Die Reform des WissZeitVG ist ein zentraler erster Hebel, mit dem sich die Weichen für eine Korrektur der aktuellen Missstände stellen lassen. Der neueste Vorschlag sieht eine Umstellung der Postdoc-Befristungshöchstdauer von derzeit sechs Jahren auf vier plus zwei Jahre vor (die letzten zwei Jahre müssen eine Anschlusszusage auf eine Entfristung enthalten) (Wiarda 2023). Diese Reform ist in ihrer aktuellen Entwurfsfassung nicht geeignet, um die notwendige fundamentale Veränderung anzustoßen (Bahr et al. 2023b). Das an sich lobenswerte Instrument der Anschlusszusage kann nur greifen, wenn diese so früh wie möglich eingesetzt wird. Eine vierjährige Postdoc-Befristung würde den wissenschaftlichen Arbeitgeber:innen das ständige Auswechseln von Postdocs weiterhin ohne größere Einschränkungen ermöglichen und zugleich einen erhöhten Druck auf die in der „Qualifikation“ befindlichen Wissenschaftler:innen ausüben; davon, dass von der Anschlusszusage unter diesen Umständen überhaupt Gebrauch gemacht wird, ist nicht auszugehen.

Eine mögliche Option, den Knoten zu lösen, wäre die Einführung einer Befristungshöchstquote, die die Wissenschaftlichen Dienste des Bundestags unlängst für verfassungskonform befunden haben (Deutscher Bundestag, Wissenschaftliche Dienste 2023). Damit würde für die einzelnen arbeitgebenden Institutionen ein konkreter Anteil festgelegt, wie viele von ihren Beschäftigten befristete Arbeitsverträge haben dürfen. Dies wäre zusammen mit der nach spätestens zwei Jahren gewährten Anschlusszusage ein effektives Mittel, um Befristung zu begrenzen, statt sie de facto in großem Stil zu ermöglichen. Eine Staffelung bei der Einführung einer Befristungshöchstquote könnte einen sukzessiven Systemübergang sicherstellen, der eine Generationengerechtigkeit garantiert, die diesen Namen auch verdient (Bahr et al. 2023a).

Das BMBF stellt sich indes auch im Hinblick auf diese tragfähige Kombination zweier aussichtsreicher Lösungsansätze stur. Nachdem das Argument der mangelnden Verfassungskonformität ausgeräumt wurde, argumentiert man nun damit, eine Befristungshöchstquote sei „nicht wissenschaftsadäquat“ (Gabel 2023). Diese Argumentation spricht Bände: Die These der fehlenden Wissenschaftsadäquatheit ist nicht mehr als eine Behauptung, für die es keinerlei Belege gibt; es fehlt schon allein an einer klaren Definition, was genau mit Wissenschaftsadäquatheit gemeint ist. Damit handelt es sich um ein Dogma ähnlich der Ineinssetzung von Innovation und Fluktuation, die ebenfalls keinerlei wissenschaftliche Grundlage hat.

Was es braucht, ist eine generelle Abkehr von einem Wissenschaftssystem, das sich nach Faktoren ausrichtet, die nachhaltige wissenschaftliche Arbeit eher verhindern als ermöglichen. Eine Umstellung der Personalstruktur hin zur unbefristeten Postdoc-Stelle als Regelfall analog zu dem, was in der sonstigen Arbeitswelt üblich ist – mit entsprechenden Weiterentwicklungs- und Aufstiegsmöglichkeiten – ist der erste Schritt hinein in eine Transformation, die in der deutschen Wissenschaftslandschaft längst überfällig ist.

Die vorliegenden Argumente, die einem Sonderbefristungsrecht das Wort reden, sind ideologisch motiviert und damit der Wissenschaft nicht würdig. Dabei ist es besonders erstaunlich, dass sich vor allem auf Lebenszeit verbeamtete oder zumindest unbefristet angestellte Personen für die ausgreifende Befristung in der deutschen Wissenschaft aussprechen – eine Befristung, unter der sie selbst nicht (mehr) zu leiden haben, andere aber sehr wohl, und die Wissenschaft gleich mit. Ein resilientes, zukunftsfähiges Wissenschaftssystem, das in einer Zeit der multiplen Krisen immer wichtiger wird, sollte nicht an fadenscheinigen Argumenten einer Lobbygruppe des mittleren und höheren Wissenschaftsmanagements scheitern. Das gilt insbesondere, da Wissenschaft „systemrelevant“ ist. Der jüngst in *Nature* festgestellten Abnahme der disruptiven Kraft von Forschungsergebnissen ist mit der Beibehaltung des Status quo schließlich nicht beizukommen (vgl. Park et al. 2023).

## Footnotes

1 Das Video wurde inzwischen von der Website des BMBF gelöscht, ist aber noch auf Youtube abrufbar: https://www.youtube.com/watch?v=PIq5GlY4h4E (27.05.2024).

2 Presseschau zum Thema hier: https://ichbinhanna.wordpress.com (27.05.2024).

3 Die Vereinigung der Kanzlerinnen und Kanzler der deutschen Universitäten hat 2019 in ihrer „Bayreuther Erklärung“ sogar das gesamte Wissenschaftssystem als „Qualifizierungssystem“ bezeichnet (Universität Bayreuth 2019).

4 Zum problematischen Zusammenhang von Wissenschaftsfreiheit und prekärer Beschäftigung siehe (Gläser & Hüther 2022).

5 Wie es um den Fachkräftemangel in der Wissenschaft bestellt ist, zeigt eindrücklich das Hochschul-Barometer 2023, aus dem hervorgeht, dass nur noch jede fünfte Hochschule ihre Personalsituation (d. h. die Ausstattung und die Rekrutierungschancen) als [eher] gut einschätzt (Hochschul-Barometer 2023: 2).

6 Hier sei daran erinnert, dass nicht nur große Mengen an unbezahlter Mehrarbeit geleistet werden, sondern das Auslaufen von Verträgen auch Mechanismen des Kündigungsschutzes (etwa bei Schwangerschaft oder Erkrankung) aussetzt, weil befristete Verträge keiner Kündigung bedürfen, um das Arbeitsverhältnis zu beenden.
